# Modeling of BN Lifetime Prediction of a System Based on Integrated Multi-Level Information

**DOI:** 10.3390/s17092123

**Published:** 2017-09-15

**Authors:** Jingbin Wang, Xiaohong Wang, Lizhi Wang

**Affiliations:** 1 School of Reliability and Systems Engineering, Beihang University, Beijing 100191, China; wangjingbin@buaa.edu.cn (J.W.); wxhong@buaa.edu.cn (X.W.); 2 Unmanned System Institute, Beihang University, Beijing 100191, China; 3 Key Laboratory of Advanced Technology of Intelligent Unmanned Flight System of Ministry of Industry and Information Technology, Beihang University, Beijing 100191, China

**Keywords:** multi-level system, lifetime prediction, Bayesian Networks, multi-sensor information integration, complex logical correlation

## Abstract

Predicting system lifetime is important to ensure safe and reliable operation of products, which requires integrated modeling based on multi-level, multi-sensor information. However, lifetime characteristics of equipment in a system are different and failure mechanisms are inter-coupled, which leads to complex logical correlations and the lack of a uniform lifetime measure. Based on a Bayesian network (BN), a lifetime prediction method for systems that combine multi-level sensor information is proposed. The method considers the correlation between accidental failures and degradation failure mechanisms, and achieves system modeling and lifetime prediction under complex logic correlations. This method is applied in the lifetime prediction of a multi-level solar-powered unmanned system, and the predicted results can provide guidance for the improvement of system reliability and for the maintenance and protection of the system.

## 1. Introduction

Life expectancy is of great significance to ensure safe and reliable operation of products, and demands for superior product performance are increasing. Devices and components with multiple lifetime characteristics are associated by complex logic to form a multi-level, complex system whose state features are difficult to quantify and predict directly. Therefore, the integration of multi-sensor information is a necessary path to achieving lifetime prediction for such systems. In fact, research on the integration of multi-sensor information is one of the trends in comprehensive utilization of data and monitoring technology innovation in the international research community. Through signal processing from multiple sensors, combined with optimization theory [1], pattern recognition [2], filtering technique [3,4], neural networks [5], artificial intelligence [6], etc., the goal is to achieve the correlation and combination of information, and provide more accurate monitoring and evaluation of products.

In order to effectively integrate equipment information provided by sensors in a complex, multi-level system to achieve evaluation and prediction of the lifetime of a system, the following requirements must be met: first, sensor data are required to carry out evaluation at the equipment level for designing prediction models. Second, the prediction framework that can integrate these low-level prediction models must be provided. A variety of prediction methods have been developed at the equipment level, including statistical methods that are based on Wiener diffusion [7], the gamma process [8], and the Markov process [9], as well as artificial intelligence methods such as data-driven neural networks [10,11] and filtering techniques [12,13], and other methods based on physical failure [14]. For instance, Nguyen considered the predictive reliability, dependencies of components and the hierarchy configuration to study the multi-level system [15]. Zheng combined unscented Kalman Filter and Relevance Vector Regression to predict the remaining useful life of lithium-ion batteries [16]. Hamed compared the stochastic simulation and the inverse First Order Reliability Method in remaining useful life prediction under different forms of uncertainty [17]. Actually, owing to evolving sensor technology, studies on prediction models for lower level objects and simple products are prevalent, and data-based lifetime prediction technology is relatively mature. However, the construction of the system prediction framework should take into account the uniform quantification of lifetime indicators and the expression of complex logic. Coolen extended the sensor status information obtained by a sensor to the probability level, and carried out the uncertainty prediction research for many types of components [18]. Jackson and Mosleh also studied the predictive modeling of uncertainty for multilayer overlapping data in complex systems [19]. The above studies affirm the advantage of a probabilistic description of complex system states and logical association uncertainties. On the other hand, George [20] and Meineri [21] adopted directed graphs in their research on complex system topology modeling and multi-factor complex correlation, which inspired the idea of modeling logical relationships of multi-level system interactions.

The Bayesian network (BN), proposed by Pearl [22], is a reasoning model based on Bayesian theory and graph theory that integrates probability and graphitization. It can be applied to make up the deficiency of traditional reliability analysis method. For an example, with the Fault Tree Analysis (FTA) method it is difficult to model systems with polymorphism problems, random uncertainties and dependent events, and some calculations are impossible to achieve due to the computation complexity under the disjointed algorithm scenario, while BN is able to describe the multi-state, uncertainties and correlations to conduct two-way inference due to probability basis, conditional independent principle and information integration skills, as well as generate results conveniently according to the available FT and simplify modeling problems. Therefore BN has a great advantage in complex system modeling applications such as disease diagnosis, financial risk analysis, and wireless sensor network and system reliability analysis, and provides the application basis for integration of multi-level information [23], which can be used to carry out the construction of lifetime prediction models based on state probability. A BN qualitative network topology and quantitative conditional probability description are endowed with strong ability to express large complex systems that have a large number of subsystems [24], which is applicable for the modeling of complex relationships such as system reliability diagrams and fault trees that are difficult to describe using traditional models [25,26]. At present, BN is widely used in the field of reliability, for system fault diagnosis [27,28], safety analysis [29], and optimization of maintenance strategy [30]. For instance, Cai et al. proposed a BN-based data-driven fault detection and diagnosis methodology which is equipped with a good toleration for sensor noise and bias of PMSM drive system [31]. Petek conducted an evaluation of failure conditional probability with a multi-level system by BN method [32]. Hu et al. developed an integrated safety prognosis model with BN to study the propagation mechanisms of faults in complex system [33]. However, there are few reports on the prediction method for a complex, multi-level system [34]. Kabir et al. investigated the failure prediction method of water mains with BN by considering the uncertainties from multi-source data and human interpretations with different credibility [35]. Therefore, system lifetime prediction based on BN to achieve the integration of equipment information from multiple sensors has substantial research value and exploration significance.

In order to solve the problem of lifetime prediction of multi-level systems under complex logic relationships and guide the design, improvement, and maintenance of systems, this paper studies the BN lifetime prediction method based on the integration of multi-level information. The system life expectancy is estimated by considering different failure mechanisms and the logical association of its mutual coupling, and the integration of equipment information with different lifetime characteristics. This paper is organized as follows: in Section 2, we present an overview of the basic concept, construction, and estimation algorithm of the BN model. In Section 3, we describe the modeling process of BN lifetime prediction and its solution method based on the prediction model at equipment level. In Section 4, we present the verification of the prediction model and in Section 5 a case study of a solar unmanned system. Finally, a summary and outlook are given in Section 6.

## 2. Overview of BN Model

A BN is a graphical network model based on the probabilistic reasoning of Bayesian theory. It consists of a Directed Acyclic Graph (DAG) and a Conditional Probability Table (CPT). The former is a graphical structure composed of directed edges that connect the node variable $X=\left\{X_{1},X_{2},\cdots ,X_{n}\right\}$ based on causality. The latter is the quantitative expression of the logical relationship of variables. The directed edge in a DAG always has the parent node pointing to the child node, while the variable with no parent node is the root node and the variable with no child node is the leaf node, and the rest are the intermediate nodes.

The probability calculation of a BN is based on the conditional independence assumption, i.e., that the probability of the child node depends only on the parent node and is independent from the other child nodes of the parent node, as shown in Equation (1). Therefore, when solving the probability of a multi-node joint, a BN only needs to consider the correlation of variables, thus reduces the solving complexity:

(1) $$P\left(X_{i}|X_{pi},X_{pai}\right)=P\left(X_{i}|X_{pi}\right)$$

where $X_{pi}$ is the parent node of node $X_{i}$ and $X_{pai}$ the child node of $X_{pi}$ other than $X_{i}$. Applying conditional independence to chain rules enables computation of the joint probability, as follows:

(2) $$P\left(X_{1},X_{2},\cdots ,X_{n}\right)=\prod_{i=1}^{n}P\left(X_{i}|X_{pi}\right)$$

The following three elements must be completed for a BN construction:

1. Determine node variables and their interpretation.
2. Create a DAG with a directed edge connecting node variables.
3. Create a CPT for non-root nodes.

**Figure 1:**
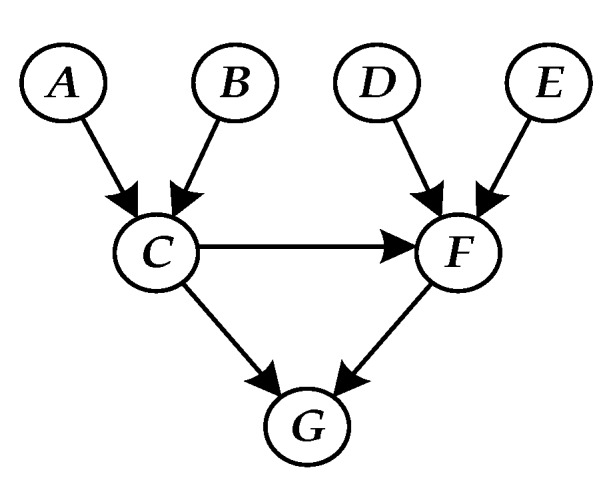
Seven-node DAG of system G.

The CPT of node G is established for the seven-node DAG of Figure 1 according to the series logic, as shown in Table 1.

**Figure 2:**
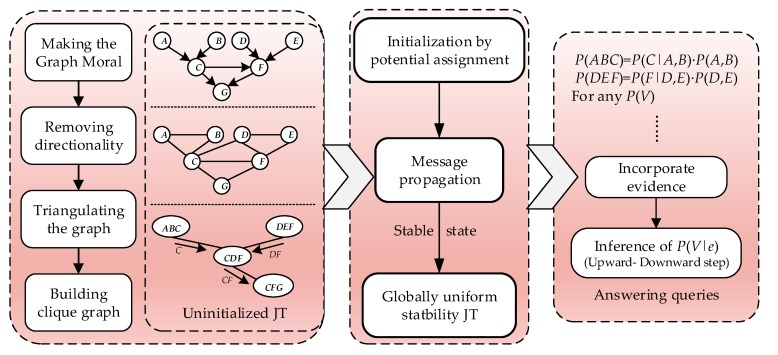
Algorithm and build thought of JT.

In the precision reasoning and approximate reasoning algorithm, the joint tree (JT) algorithm based on clique tree propagation is widely used because of its unique advantages of high search efficiency, capability of returning logarithmic results, and dual-channel transmission. The solution is shown in Figure 2. The JT is obtained by the steps of the transformation and triangulation of the DAG. After initialization, the nodes can absorb the information and update the distribution function $\phi_{C}$ of the partition nodes to achieve information transmission. When the single-transfer process of the JT information satisfies the globally consistent steady state, the distribution of V can be obtained according to $P\left(V\right)=\sum_{C\left\{V\right\}}\phi_{C}$ for any desired variable V. When new evidence e is added, the conditional probability distribution of the variable V is solved as follows:

(3) $$P\left(V|e\right)=\frac{P\left(V,e\right)}{P\left(e\right)}=\frac{P\left(V,e\right)}{\sum_{V}P\left(V,e\right)}.$$

## 3. Lifetime Prediction Method of Multi-level System Based on BN

### 3.1. Prediction Model at Equipment Level

From the prediction tools and means, the prediction model at the equipment and component levels can be divided into three categories: stochastic process based, data based, and physical failure based. The optimal prediction method is chosen to establish the lifetime prediction model for root nodes of the BN, and the lifetime prediction of intermediate nodes and leaf nodes can then be deduced. Therefore, the soundness and accuracy of the model and parameters of the equipment can affect the prediction of higher-level nodes.

The system of a complex product often involves performance degradation and accidental failure. Here, the equipment whose performance degradation obeys the Wiener process is taken as an example to devise a prediction model. Assuming that the performance parameter W is a key indicator of product lifetime and is sensitive to stress S, the parameter then follows the Wiener degradation process as follows:

(4) $$W\left(t\right)=\mu \left(s\right)\cdot t+\sigma \cdot B\left(t\right)+W_{0}$$

where W(t) is the product performance at time t, and μ(s) is the drifting coefficient reflecting the performance degradation rate, which is a function of stress and time. In an accelerated model, $\mu \left(s\right)=\exp\left[\beta_{0}+\beta \varphi \left(s\right)\right]$. Constant σ is the diffusion coefficient that is irrelevant with respect to environment and time. B(t)~N(0,t) is the standard Brownian motion and $W_{0}$ is the initial value of the parameter.

The degradation amount within the time Δt from the properties of the Wiener process is $\Delta W\sim N\left(\mu \left(s\right)\Delta t,\sigma^{2}\Delta t\right)$. L is defined as the failure threshold of performance W, and then the time $t^{\prime }$ that the performance parameter value first passed through L satisfies the inverse Gaussian distribution. The distribution function is the unreliability function of the product, and the corresponding probability density function is given by:

(5) $$f\left(t;W_{0},L\right)=\frac{L-W_{0}}{\sqrt{2\pi \sigma^{2}t^{3}}}\exp\left\{-\frac{{\left[\left(L-W_{0}\right)-\mu \left(s\right)t\right]}^{2}}{2\sigma^{2}t}\right\}$$

The corresponding reliability function is the prediction model of equipment lifetime, as follows:

(6) $$R\left(t\right)=\Phi \frac{\left(L-W_{0}\right)-\mu \left(s\right)t}{\sigma \sqrt{t}}-\exp\left\{\frac{2\mu \left(s\right)L}{2\sigma^{2}}\right\}\Phi \frac{\left(L-W_{0}\right)+\mu \left(s\right)t}{\sigma \sqrt{t}},$$

where Φ (*) is the cumulative distribution function of the standard normal distribution.

Other prediction methods and models based on the data and physical failure can also provide the corresponding prediction information of product lifetime under the timescale satisfying prediction accuracy.

### 3.2. BN Prediction Modeling and Inference at System Level

Differences exist in the lifetime characteristics of different products. Therefore, the state probability of the unified lifetime based on quantitative indicators is used in this paper. For example, for the case of accidental hardware failure, reliability and cumulative probability of failure can describe the probability in the “normal” and “fault” states. However, for a degradation mechanism in which the lifetime characteristics cannot be directly described by the reliability, the complete state set must be customized before modeling to describe the “intact degree” and “failure degree” as two opposing events, to ensure that the sum of the state probability is 1. For ease of expression, in this work we use ℛ to characterize the “intact” state of all nodes, with corresponding probability R, and represent the corresponding “failure” state with ℱ, with a probability of F. 

**Figure 3:**
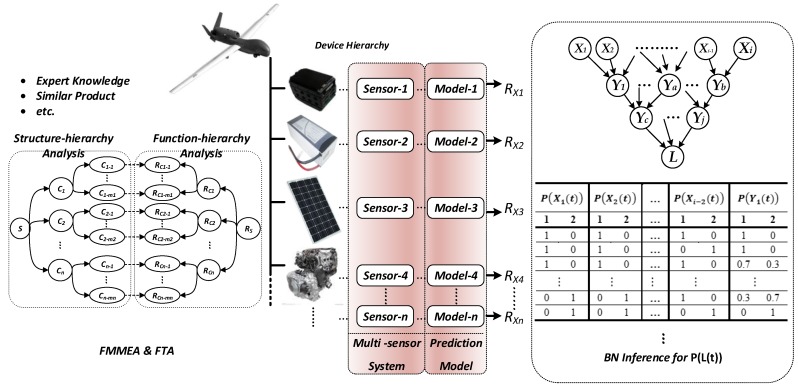
Modeling process of BN prediction at system level based on integrated multi-sensor information.

The lifetime prediction sequence of state probability reflecting lifetime information given by each equipment prediction model is taken as the prior probability of the corresponding root node in the system BN, and the probability of unknown nodes is deduced to achieve the integration and prediction of the lifetime information from the multi-sensor (process shown in Figure 3). The specific model construction proceeds via the following steps:

1. Obtain expert knowledge and structure and function information of similar products. Analyze the failure mode and mechanism through the system function-level method. Fault tree analysis is used for key faults to determine the equipment and mechanisms that affect system lifetime.
2. Deploy sensors for key performance parameters of each device. Collect and process data.
3. Based on the analysis of sensor data, the prediction model is established for each piece of equipment, and the prediction value of lifetime-related state probability is given.
4. Combined with the system failure mechanism, the prediction value of state probability for the equipment involved is used as the prior probability for the root node.
5. Combine system logic to form a DAG, and establish a non-root node CPT.
6. The JT estimation algorithm is used to solve the joint probability of relevant nodes, to update the conditional probability values of each node, and to achieve the deduction of state probability of system nodes to complete the system prediction.
7. The BN prediction model is still applicable, along with the updating of sensor data and correction of prediction model at equipment level. If the failure mechanism changes, the DAG and CPT should be corrected for the updated logical relationship; proceed to Step 3.

In the above BN model that interprets node variable with state probability, $S_{X_{i}}^{a_{i}}\left(t\right),\;S_{Y_{j}}^{b_{j}}\left(t\right)$, and $S_{L}^{k}\left(t\right)$ are used to represent the $a_{i},\;b_{j},\;k\;\mathrm{th}$ state of root node $X_{i}\;\left(i=1,2,\cdots ,p\right)$, the intermediate node $Y_{j}\;\left(j=1,2,\cdots ,q\right),$ and the leaf node L at time t respectively, i.e., $a_{i}=b_{j}=k=1,2$.

For a number of p root nodes, the probability of the state ℛ is solved by the prediction model for each respective piece of equipment and discretized according to the unsupervised equal-width interval method with a time sequence to achieve the state prediction in the future T time; that is, the p×n-order state probability prediction matrix:

$$R_{p\times n}=\left(\begin{array}{ccc} R_{1,1} & \cdots & R_{1,n}\\ \vdots & \ddots & \vdots \\ R_{p,1} & \cdots & R_{p,n} \end{array}\right),$$

with a certain time sequence T=(t+ζ,t+2ζ,⋯,t+nζ). The elements $R_{i,\tau }=R\left(X_{i}\left(\tau \right)\right)\;\left(i=1,2,\cdots ,p;\;\tau =1,2,\cdots ,n\right)$ represent the probability of the device $X_{i}$ to be in the state R. If ζ is taken as the unit time, the probability set $R\left(t\right)={\left(R_{1,t},R_{2,t},\cdots ,R_{p,t}\right)}^{T}$ corresponds to the probability of p nodes to be in set X at state ℛ. Correspondingly, the probability of occurrence of state F is given by F(t)=1−R(t).

When the abovementioned multi-sensor information is used to deduce the lifetime prediction based on the BN, the probability of the root node is firstly assigned according to the probability prediction matrix. The probability of the root node $X_{i}$ at time t is $P\left(S_{X_{i}}\left(t\right)\right)=R\left(X_{i}\left(t\right)\right),i=1,2,\cdots ,p$. For the solution of the state probability of intermediate nodes, it is assumed that the parent-node set $X=\left\{X_{1},X_{2},\cdots ,X_{i}\right\}$ exists for the node $Y_{j}$. According to the assumption of independent conditions, the probability prediction of the intermediate nodes at time t can be solved based on:

(7) $$\begin{array}{ll} P\left(Y_{j}\left(t\right)\right) & =\sum_{X}P\left(S_{Y_{j}}\left(t\right),S_{X}\left(t\right)\right)\\ & =\sum_{X}P\left(S_{Y_{j}}^{2}\left(t\right)|S_{X}\left(t\right)\right)P\left(S_{X_{1}}\left(t\right)\right)\cdots P\left(S_{X_{1}}\left(t\right)\right)\;, \end{array}$$

**Figure 4:**
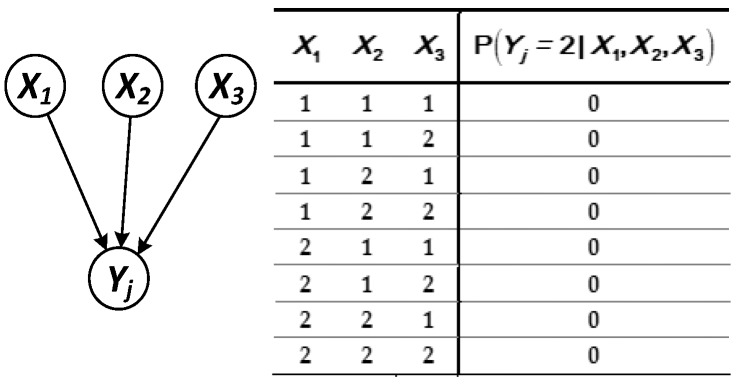
Logical structure in which the parent node is the root node.

The complexity of logical associations of the BN will increase with the number of nodes contained. Based on the advantages of conditional independence, the complex topological equivalence can be divided into simple structures, and the construction and inference of three kinds of basic structural prediction models are taken as examples as follows:

(1) The parent node set $X=\left\{X_{1},X_{2},X_{3}\right\}$ of node $Y_{j}$ contains only the root node (Figure 4). 

Each state of the parent node is independent of each other at any time, and then the predicted probability of $Y_{j}$ in state ℛ at time t is as follows:

(8) $$\begin{array}{ll} R\left(Y_{j}\left(t\right)\right) & =\sum_{X_{1},X_{2},X_{3}}P\left(S_{Y_{j}}\left(t\right),S_{X_{1}}\left(t\right),S_{X_{2}}\left(t\right),S_{X_{3}}\left(t\right)\right)\\ & =\sum_{X_{1},X_{2},X_{3}}P\left(S_{Y_{j}}^{2}\left(t\right)|S_{X}\left(t\right)\right)P\left(S_{X_{1}}\left(t\right)\right)P\left(S_{X_{2}}\left(t\right)\right)P\left(S_{X_{3}}\left(t\right)\right)\\ & =P\left(S_{Y_{j}}^{2}\left(t\right)|S_{X_{1}}^{2}\left(t\right),S_{X_{2}}^{2}\left(t\right),S_{X_{3}}^{2}\left(t\right)\right)P\left(S_{X_{1}}^{2}\left(t\right)\right)P\left(S_{X_{2}}^{2}\left(t\right)\right)P\left(S_{X_{3}}^{2}\left(t\right)\right)\;. \end{array}$$

**Figure 5:**
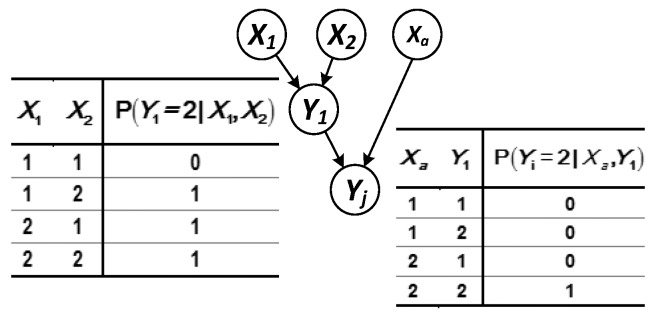
Logical structure in which the parent node contains both the intermediate and root nodes.

(2) The parent node set $X=\left\{X_{a},Y_{1}\right\}$ of node $Y_{j}$ contains both the root node and the intermediate node (Figure 5).

The predicted probability of node $Y_{j}$ at time t can be solved according to:

(9) $$\begin{array}{ll} R\left(Y_{j}\left(t\right)\right) & =\sum_{Y_{1},X_{a}}P\left(S_{Y_{j}}^{2}\left(t\right)|S_{Y_{1}}^{2}\left(t\right),S_{X_{a}}^{2}\left(t\right)\right)P\left(S_{X_{a}}^{2}\left(t\right)\right)\sum_{X_{1},X_{2}}P\left(S_{Y_{1}}^{2}\left(t\right)|S_{X_{1}}\left(t\right),S_{X_{2}}\left(t\right)\right)\cdot P\left(S_{X_{1}}\left(t\right)\right)P\left(S_{X_{2}}\left(t\right)\right)\\ & \;=P\left(S_{Y_{j}}^{2}\left(t\right)|S_{Y_{1}}^{2}\left(t\right),S_{X_{a}}^{2}\left(t\right)\right)P\left(S_{X_{a}}^{2}\left(t\right)\right)\cdot [P\left(S_{Y_{1}}^{2}\left(t\right)|S_{X_{1}}^{2}\left(t\right),S_{X_{2}}^{2}\left(t\right)\right)P\left(S_{X_{1}}^{2}\left(t\right)\right)P\left(S_{X_{2}}^{2}\left(t\right)\right)\\ & +P\left(S_{Y_{1}}^{2}\left(t\right)|S_{X_{1}}^{1}\left(t\right),S_{X_{2}}^{2}\left(t\right)\right)P\left(S_{X_{1}}^{1}\left(t\right)\right)P\left(S_{X_{2}}^{2}\left(t\right)\right)+P\left(S_{Y_{1}}^{2}\left(t\right)|S_{X_{1}}^{2}\left(t\right),S_{X_{2}}^{1}\left(t\right)\right)P\left(S_{X_{1}}^{2}\left(t\right)\right)P\left(S_{X_{2}}^{1}\left(t\right)\right)]\;. \end{array}$$

**Figure 6:**
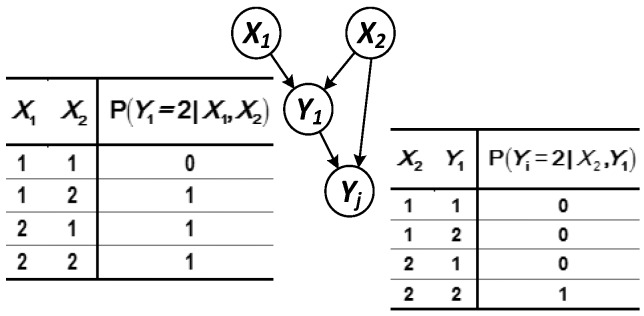
Logical structure in which the same parent node points to multiple subnodes.

(3) There is a case in which the same parent node points to multiple child nodes (see Figure 6).

The system model that has interconnected logical correlation mostly has network structure. The same parent node pointing to multiple sub-nodes is the basis for the composition of the network structure. This situation can be predicted according to:

(10) $$\begin{array}{ll} R\left(Y_{j}\left(t\right)\right) & =\sum_{X_{2},Y_{1}}P\left(S_{Y_{j}}^{2}\left(t\right)|S_{X_{2}}\left(t\right),S_{Y_{1}}\left(t\right)\right)\sum_{X_{1},X_{2}}P\left(S_{Y_{1}}\left(t\right)|S_{X_{1}}\left(t\right),S_{X_{2}}\left(t\right)\right)\cdot P\left(S_{X_{1}}\left(t\right)\right)P\left(S_{X_{2}}\left(t\right)\right)\\ & =P\left(S_{Y_{j}}^{2}\left(t\right)|S_{X_{2}}^{2}\left(t\right),S_{Y_{1}}^{2}\left(t\right)\right)\cdot [P\left(S_{Y_{1}}^{2}\left(t\right)|S_{X_{1}}^{2}\left(t\right),S_{X_{2}}^{2}\left(t\right)\right)P\left(S_{X_{1}}^{2}\left(t\right)\right)P\left(S_{X_{2}}^{2}\left(t\right)\right)\\ & +P\left(S_{Y_{1}}^{2}\left(t\right)|S_{X_{1}}^{1}\left(t\right),S_{X_{2}}^{2}\left(t\right)\right)P\left(S_{X_{1}}^{1}\left(t\right)\right)P\left(S_{X_{2}}^{2}\left(t\right)\right)]\;. \end{array}$$

Based on the probability of the root node and intermediate node, the predicted probability of the leaf node in state R can be further solved according to:

(11) $$\begin{array}{ll} R_{L}\left(t\right) & =\sum P\left(S_{X_{1}}\left(t\right),S_{X_{2}}\left(t\right),\cdots ,S_{X_{p}}\left(t\right),S_{Y_{1}}\left(t\right),S_{Y_{2}}\left(t\right),\cdots ,S_{Y_{q}}\left(t\right),S_{L}^{2}\left(t\right)\right)\\ & =\sum_{Pa\left(L\right)}P\left(S_{L}^{2}\left(t\right)|S_{Pa\left(L\right)}\left(t\right)\right)\cdot \sum_{Pa\left(Y_{1}\right)}P\left(S_{Y_{1}}\left(t\right)|S_{Pa\left(Y_{1}\right)}\left(t\right)\right)\cdots \\ & \sum_{Pa\left(Y_{q}\right)}P\left(S_{Y_{q}}\left(t\right)|S_{Pa\left(Y_{q}\right)}\left(t\right)\right)\cdots P\left(S_{X_{1}}\left(t\right)\right)\cdots P\left(S_{X_{p}}\left(t\right)\right)\;, \end{array}$$

where Pa(∗) is the parent node of the node “(∗)”.

According to the above formula, the JT estimation algorithm traverses the DAG, and the state and lifetime of the system node L can be predicted. By the probability prediction matrix $R_{p\times n}=\left(R\left(t\right),R\left(t+\zeta \right),R\left(t+2\zeta \right),\cdots ,R\left(t+2\zeta \right)\right)$ of the root node, the corresponding prediction sequence of probability at system level will be obtained to achieve continuous prediction of the lifetime.

## 4. Modeling, Simulation, and Verification of System-Level BN Prediction

The seven-node DAG shown in Figure 1 contains the three basic structures described above, and the BN prediction method is simulated and verified as an example. The three-tier system G consists of parallel subsystems C and F, comprising four devices A, B, D, and E. In the subsystem C(F), A (D) and B (E) conduct different functions, respectively, which are in series logic. In addition, according to the early collection of product information, the logic that “if the state of subsystem C is abnormal, there is a 60% probability of failure for subsystem F” exists between the subsystems. The key performance parameters of each piece of equipment were determined, monitoring devices were arranged, and the sensor signals were extracted, processed, and analyzed. The lifetime prediction model of each piece of equipment was then obtained. 

(1) The lifetime characteristics of equipment A and B meet the accidental failure, while D and E meet the degradation failure, and the corresponding prediction models are shown in Table 2.

Based on the prediction model above, the BN model with both accidental failure and degeneration failure is established to predict the system lifetime of the next 2000 h. The state probability of system G can be solved by:

(12) $$\begin{array}{ll} R_{G}\left(t\right) & =\sum P\left(S_{A}\left(t\right),S_{B}\left(t\right),S_{C}\left(t\right),S_{D}\left(t\right),S_{E}\left(t\right),S_{F}\left(t\right),S_{G}^{2}\left(t\right)\right)\\ & =\sum_{C,F}P\left(S_{G}^{2}\left(t\right)|S_{C}\left(t\right),S_{F}\left(t\right)\right)\cdot \sum_{A,B}P\left(S_{C}\left(t\right)|S_{A}\left(t\right),S_{B}\left(t\right)\right)\\ & \;\sum_{C,D,E}P\left(S_{F}\left(t\right)|S_{D}\left(t\right),S_{E}\left(t\right),S_{C}\left(t\right)\right)P\left(S_{A}\left(t\right)\right)P\left(S_{B}\left(t\right)\right)P\left(S_{D}\left(t\right)\right)P\left(S_{E}\left(t\right)\right). \end{array}$$

**Figure 7:**
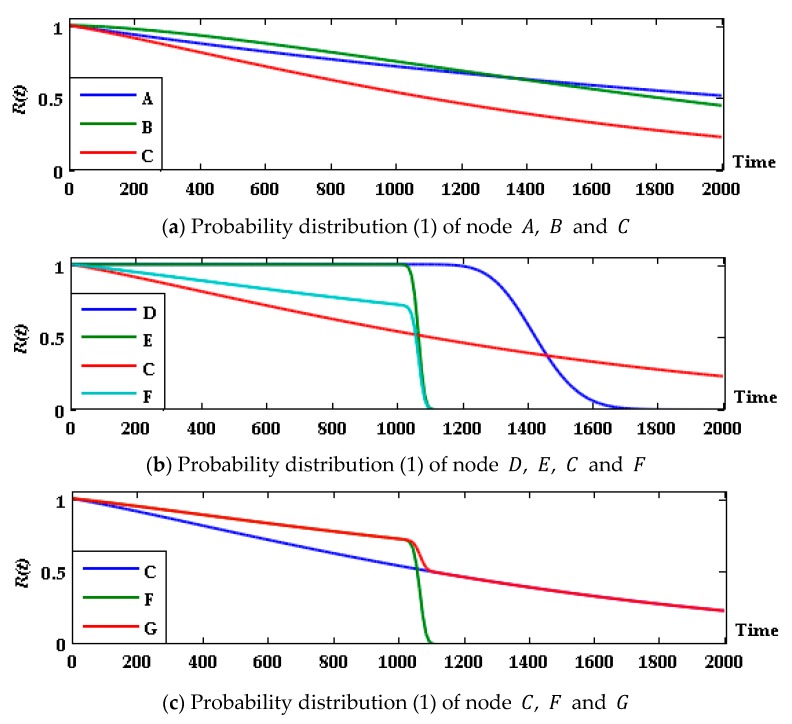
Probability distribution (1) of parent and child nodes at state R for G system group.

Due to the efficient two-way accurate-inference ability, “engine = jtree _ inf _ engine (bnet)” of the Bayes Net Toolbox (BNT) for MATLAB developed by Murphy [36] is used to conduct inference, and the probability of each group of parent and child nodes in state R is shown in Figure 7.

The results show that in the first 1000 h the subsystem F shows better performance than C, and the state probability of system G is the same as that of the former. After 1030 h, however, with the performance degradation, the failure probability of F suddenly increases, and the probability of system G being in state R decreases. After 1090 h, subsystem F completely failed, and the system state was completely determined by subsystem C. Therefore, the prediction curve trends of C and G after this point are basically the same. The lifetime prediction of the system is estimated and evaluated: the median life of the system $t_{0.5}=1098\;h$; if the system in good condition has a probability threshold of 0.45, the remaining life is approximately 1226 h.

**Figure 8:**
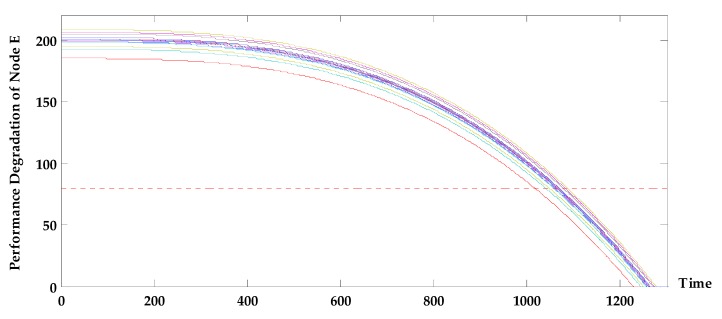
Simulation curve of performance degradation of device E..

**Figure 9:**
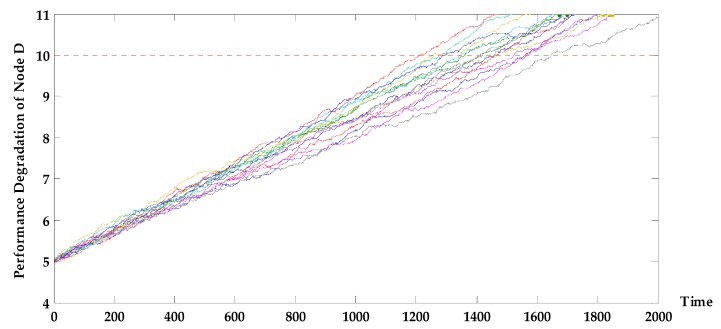
Simulation curve of performance degradation of device D.

In addition, the performance degradation simulation curve of E (as shown in Figure 8) shows that from 1010 to 1090 h the performance parameters of all 15 samples degenerated below the threshold of 80, and that the 15 samples of device D did not exceed the performance parameter threshold in the first 1200 h (a simulated degradation curve is shown in Figure 9). Through the analysis of the subsystem F, we can see that the main reason for the sudden deterioration of the system is the performance degradation of device E under cyclic stress, which caused it to exceed the performance threshold with considerable probability.

The dotted lines parallel to the horizontal axis of Figure 8 and Figure 9 represent the device performance threshold.

(2) For all equipment that is in degradation failure mode, the corresponding prediction model and parameters are shown in Table 3.

**Figure 10:**
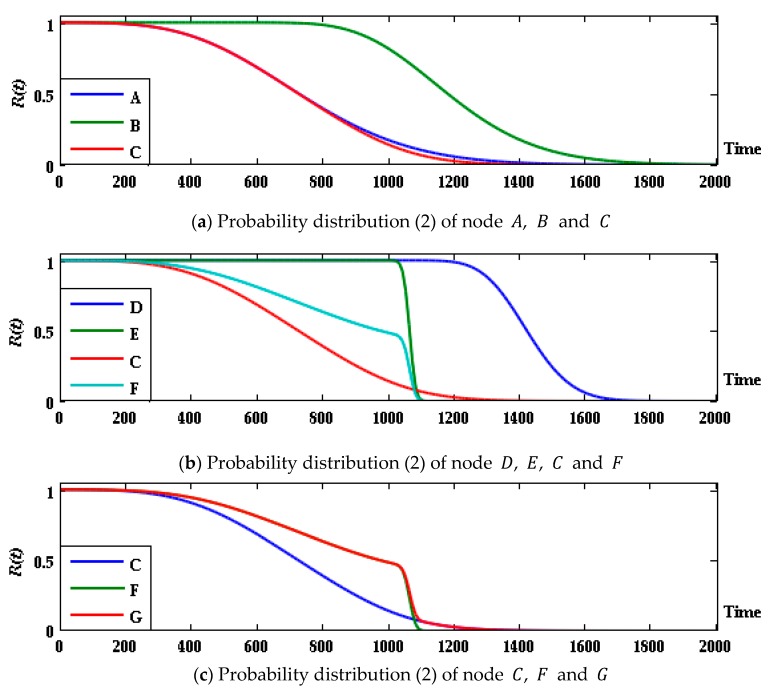
Probability distribution (2) of parent and child nodes at state R for G system group.

The state probability prediction curve of each (sub-) system is obtained and shown in Figure 10.

**Figure 11:**
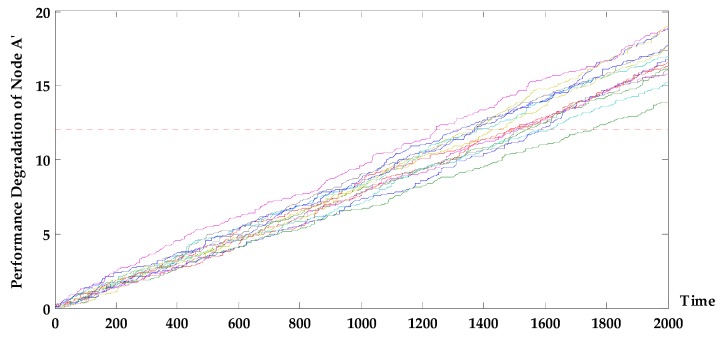
Simulation curve of performance degradation of equipment $A^{\prime }$.

Compared to the predicted result above, a system whose devices are all in degradation failure mode has a low probability of staying intact at 1030 h, specifically, only 0.622. However, the state probability predicted above at 1030 h is 0.712, and the working state was maintained with a probability of 0.46 until 1200 h. With a probability threshold of 0.45, the remaining life of the system will be less than 1064 h. The main reason for the change of system state is subsystem C, in which the performance parameter of device A follows the Gamma degradation process (Figure 11). The probability of device A to be at state 2 is reduced to 0.5224 at 1000 h, making the system state significantly degraded; after this prediction point, the performance degradation of device E in subsystem F is deteriorated, and the system fails at a faster rate. On one hand, subsystem C has direct effect on the state of system G. On the other hand, the state of G is indirectly affected through the correlation with subsystem F. Therefore, the degradation rate of a system state after integration is faster.

It can be verified through the above simulation that a BN can achieve the integration of lifetime information from multiple sensors in a multi-level system when the equipment grade product experiences accidental failure or (and) degradation failure, which reasonably predicts system lifetime from the perspective of state probability, and shows good compatibility and integration for a variety of low-level prediction models.

## 5. Application Case

### 5.1. BN Lifetime Prediction of an Unmanned Solar-Powered System

An unmanned system is a typical multi-level system with complex structure and function logic. With the air-ground collaborative work requirements, the system achieves its functional requirements and sharing of information resources layer by layer. At the same time, under environmental influences such as temperature, humidity, vibration, and interference the unit with the degradation property and that with the accidental failure together affect the system state and lifetime. In this paper, a solar-powered unmanned aerial vehicle (UAV) system was chosen as an example to carry out lifetime prediction based on a BN.

After the system is simplified, the hardware is mainly composed of an airborne system, a data link, and a ground system. The energy system inside the airborne system must provide power support for the normal operation of the navigation system, flight control system, power system, communication system, etc., and it is subject to the state of the battery management system and the solar panel. Any abnormality can cause the energy system to degrade or even fail to function properly.

**Figure 12:**
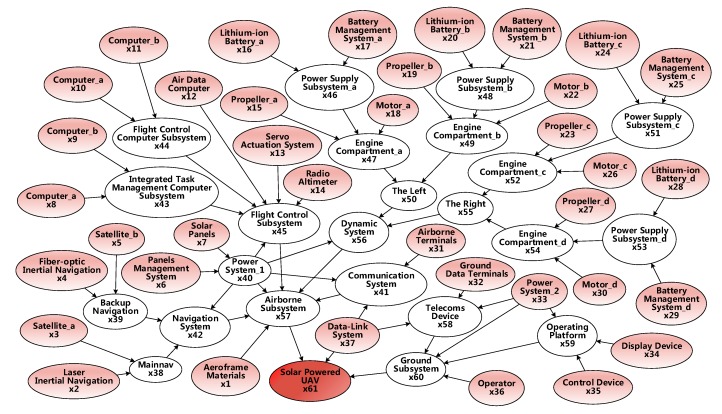
DAG of a solar-powered unmanned system.

The navigation system contains the master and lead. A flight control computer subsystem for integrated task management, a flight control computer subsystem, and a servo action system are necessary. In addition to the airborne terminal, the airborne communication system must go through the data link to achieve the transmission and exchange of information with ground communication equipment. In the ground subsystem, the power supply system is the energy supply system for flight operation, the console, communication equipment, and other equipment. For the console, the requirement is to simultaneously display and control functions. This BN model fully accounts for the relevance of intermediate nodes. In these subsystems, degradation failure exists in the solar cell and UAV body material, which is modeled with Wiener process and Gamma process, respectively, to establish the performance degradation for lifetime prediction. Specifically, the system DAG is shown in Figure 12, and the 61 nodes contain 37 device-level nodes.

**Figure 13:**
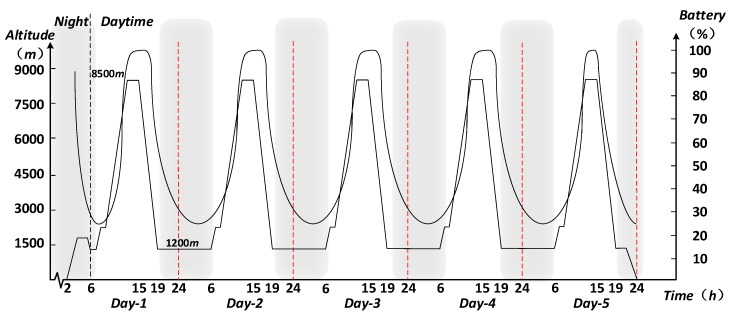
Correspondence between flight height profile of UAV and charge-discharge cycle.

At the same time, considering the characteristics of photovoltaic energy, a special flight profile is used: To ensure the full storage of the solar panels, before every sunrise the aircraft must climb from an altitude of approximately 1000 m to a cruising altitude to 8500 m. In order to reduce energy consumption, a fully charged UAV descends to a low-altitude region in the evening to take advantage of lower air resistance and will continue to fly. The ideal charge-discharge process under the flight height profile is shown in Figure 13.

**Figure 14:**
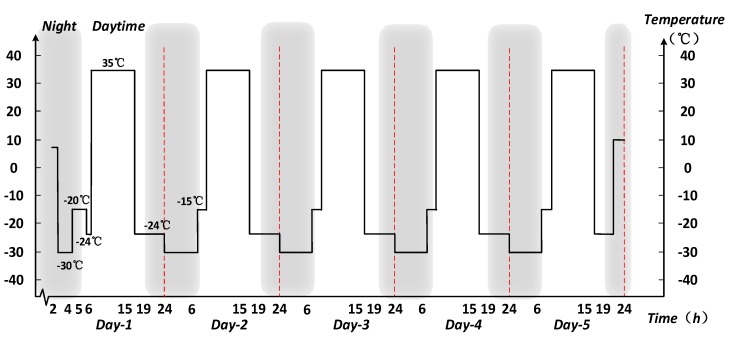
Simplified temperature profile of solar-powered UAV flight.

During the process, the ambient temperature will change cyclically with flight height, and the structure with the temperature degradation characteristic will experience significant state change. A simplified temperature stress profile is given in Figure 14.

**Figure 15:**
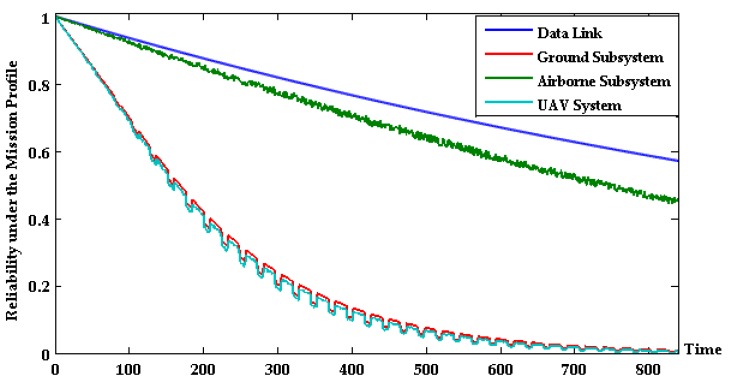
Probability prediction curve of unmanned system state.

The lifetime prediction model considering the temperature stress is established by combining the sensor information of the 37 devices, and the state of the cruise mission in the next 35 d is predicted according to the above temperature profile. Figure 15 shows the state probability distribution of the solar-powered unmanned system and its data link, ground subsystem, and airborne subsystem.

The continual decreasing trend reflects the degradation of operation state of the (sub-) system with time and environmental conditions; the UAV system and airborne subsystem show cyclically violent state changes after flying 100 h approximately. This is because the temperature degradation characteristics of the airborne equipment are subject to periodic ambient temperature stress.

### 5.2. Analysis Based on the Prediction Results

(1) Task risk analysis

**Figure 16:**
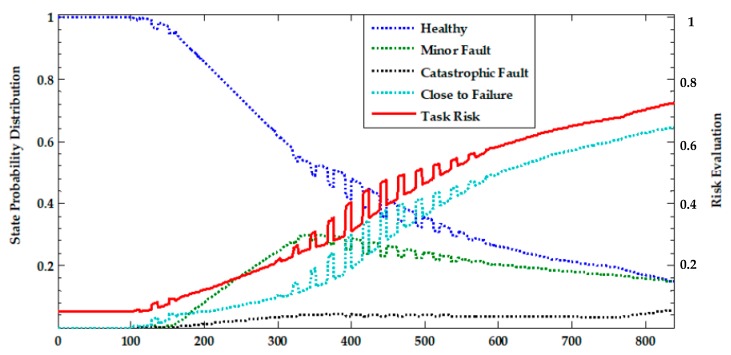
Probability distribution and risk prediction of each state of unmanned system.

In order to make full use of the predicted information to identify the health of the UAV system, a task risk analysis was carried out on the system based on health status. Based on fuzzy theory, the probabilistic predictive values for each time are divided into four healthy states, such as “healthy”, “minor fault”, “catastrophic fault”, and “close to failure” with the trapezoidal fuzzy number. The probability distribution curves of four states are shown as dotted lines in Figure 16. The risk of each state can be weighted to achieve the risk assessment of system at all times (as shown in Figure 16).

**Figure 17:**
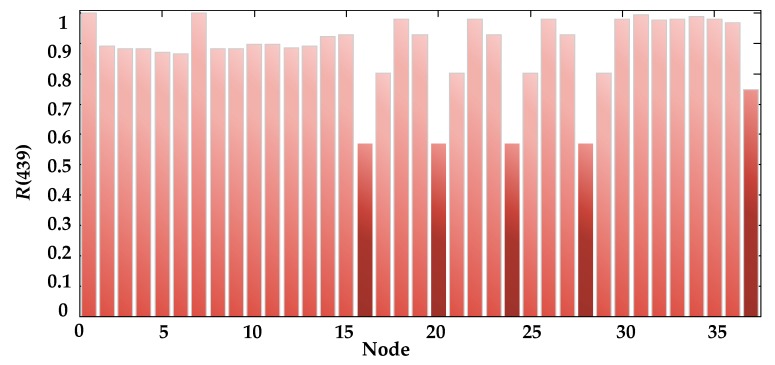
Reliability prediction of each piece of equipment at 439 h.

It can be seen from Figure 16 that the UAV system can work normally in the first 438 h, but frequent abnormal jumps between “healthy” and “close to failure” occurred between 439 and 496h, and the worst health state was maintained after 497 h, which leads to a continuous increase in systemic risk, especially after 512 h when the task risk has exceeded 0.5. Continuing to perform the task poses a considerable probability of failure and safety risks for personnel and equipment. Additionally, at the time of 439 h when state mutation firstly occurs, the reliability of each device is predicted and shown in Figure 17. 

It can be seen from the figure that the probability that the lithium battery and the data link (nodes 16, 20, 24, 28, and 37) can work normally is relatively low when the system state begins to appear abnormally, which may be the fundamental factor causing the deterioration of the system state.

(2) Sensitivity analysis of nodes

Sensitivity can be used to reflect the sensitivity of system output to the amount of input change so that the weak parts of the system can be identified. Due to the conditional independence assumption in BN, nodes at the same level are independent of each other, and thus the sensitivity of each node to the system is defined as:

(13) $$\Theta_{i,S}=\frac{\partial R_{s}}{\partial R_{X_{i}}}=\frac{\Delta R_{s}}{\Delta R_{X_{i}}}$$

**Figure 18:**
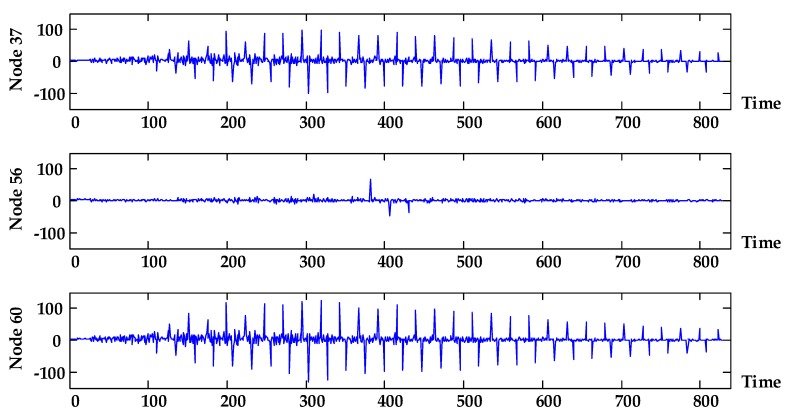
System sensitivity to three subsystems.

In the BN model of the UAV system, the sensitivity of each subsystem is shown in Figure 18.

According to the figure, the system is more sensitive to the state change of node 60 (airborne subsystem). Further analysis of the airborne subsystem shows that the sensitivity of nodes 16, 20, 24, and 28 can reach 14 orders of magnitude at a certain local time (the figures of which are not shown in this paper). In addition, node 37 (data link) is both a subsystem and root node whose sensitivity is at a high level. As a result, the airborne subsystem and data link subsystem will have a significant impact on the system state.

(3) Recommendations for reliability design and maintenance

In reliability design, sensitivity analysis can be used to improve the inherent reliability of critical equipment by design improvement. Meanwhile, close attention should be paid to redundancy design within the allowable range of aircraft load. This will create significant improvements in the reliability, availability, and longevity of UAV systems. From the point of view of maintenance and repair, the maintenance strategy $T\left(C,A^{*},P_{risk}|t\right)$ related to maintenance cost C(t), instantaneous availability $A^{*}\left(s\right)$ and work risk $P_{risk}\left(t\right)$ can be formulated according to the given lifetime index. Combined with system mission risk and the average cost of preventive and post-maintenance, a maintenance cost-time model can be established. Maintenance or replacement activities are carried out at higher cost-efficiency-ratio point in accordance with the priority of the equipment status and sensitivity.

## 6. Conclusions

In this paper we propose a modeling method for lifetime prediction of systems from integrated multi-sensor information at device level based on a BN in order to conduct system-level lifetime prediction for complex, multi-sensor systems. The simulation results show that the method can effectively solve the complex logical association of data and the quantitative description of the uncertainty in the multi-level system, and that it has good integration ability and compatibility with various prediction models of devices. At the same time, the application research of a multi-level solar-powered UAV system with multi-device information is carried out, and the task risk and node sensitivity analysis are conducted according to the prediction results of system lifetime based on the temperature profile. The research shows that the system prediction method from the integrated multi-sensor lifetime based on a BN has the following advantages:

(1) By making full use of multi-sensor information, data association, and quantitative expression, integration and lifetime prediction can be achieved on multi-level, complex, dynamic, multi-source logic data.
(2) The diversified life expectancy information of the model output is based on different aspects and complement each other, which provides comprehensive data support and theoretical guidance for design improvements and maintenance from system view and in the entire lifecycle process that considers a trade-off of economics, technology, risk, and effectiveness.

However, simulation and application studies show that the proposed method has a high dependence on the prediction model at the device level. The more accurate lifetime prediction for a complex multi-level system is based on accurate device-level prediction information, which imposes higher requirements on information acquisition, processing, and analysis from multiple sensors. Therefore, there might be limitations in practice by only considering data from sensors. Future work on lifetime prediction methods based on a BN should focus on the study and understanding of prediction accuracy, and appropriately take into account other sources and levels data like experimental data and reasonable expert knowledge and so on to conduct integration. In addition, the prediction dimension can be extended from two states to multiple states, and the intermediate process of various mechanisms can be studied in depth by considering the intermediate state to obtain more abundant lifetime prediction information.

## Figures and Tables

**Table 1:** CPT of node G .

C		F		G	
F	T	F	T	F	T
1	0	1	0	1	0
1	0	0	1	0	1
0	1	1	0	0	1
0	1	0	1	0	1

**Table 2:** Prediction model (1) of four pieces of equipment.

Node	Description of Prediction Model	Lifetime Prediction Model
A	Exponential distribution	$R_{A}\left(t\right)=\exp\left(-t/3000\right)$
B	Weibull distribution	$\;R_{B}\left(t\right)=\exp{\left[-\left(t/2300\right)\right]}^{1.5}$
D	Wiener degradation process; Drift parameters are selected as the Arrhenius model	$R_{D}\left(t\right)=-\exp\left[\frac{2d\left(s\right)\left(L-Y_{0}\right)}{\sigma^{2}}\right]\Phi \left(-\frac{L-Y_{0}+d\left(s\right)t}{\sigma \sqrt{t}}\right)+\Phi \left(\frac{L-Y_{0}-d\left(s\right)t}{\sigma \sqrt{t}}\right)$ $\left(Ea_{D}=0.473,A_{D}=4.7\times 10^{5};Y_{0-D}=5,L_{D}=10,\sigma_{D}=0.01\right)$
E	Degradation under cyclic stress	$R_{E}\left(t\right)=\Phi \left(\left(-0.0001{\left(t/10\right)}^{3}+200-80\right)/\sqrt{5}\right)$

**Table 3:** Prediction model (2) of four pieces of equipment ^1^.

Node	Description of Prediction Model	Lifetime Prediction Model
$A^{\prime }$	Gamma process; Scale parameters are selected as the Arrhenius model	$R_{A^{\prime }}\left(t\right)=\Gamma \left(v\left(s\right)t,L/u\right)/\Gamma \left(v\left(s\right)t\right)$ $\left(Ea_{A^{\prime }}=0.453,A_{A^{\prime }}=3.9\times 10^{5};Y_{0-A^{\prime }}=0,L_{A^{\prime }}=12,u_{A^{\prime }}=2.3\right)$
$B^{\prime }$	Wiener process	$\left(Ea_{B^{\prime }}=0.473,A_{B^{\prime }}=6.7\times 10^{5};Y_{0-B^{\prime }}=6,L_{B^{\prime }}=12,\sigma_{B^{\prime }}=0.032\right)$

^1^ Prediction models of D and E are the same as in Table 3.
